# Predicting Cortical Dark/Bright Asymmetries from Natural Image Statistics and Early Visual Transforms

**DOI:** 10.1371/journal.pcbi.1004268

**Published:** 2015-05-28

**Authors:** Emily A. Cooper, Anthony M. Norcia

**Affiliations:** Department of Psychology, Stanford University, Stanford, California, United States of America; Technische Universitat Chemnitz, GERMANY

## Abstract

The nervous system has evolved in an environment with structure and predictability. One of the ubiquitous principles of sensory systems is the creation of circuits that capitalize on this predictability. Previous work has identified predictable non-uniformities in the distributions of basic visual features in natural images that are relevant to the encoding tasks of the visual system. Here, we report that the well-established statistical distributions of visual features -- such as visual contrast, spatial scale, and depth -- differ between bright and dark image components. Following this analysis, we go on to trace how these differences in natural images translate into different patterns of cortical input that arise from the separate bright (ON) and dark (OFF) pathways originating in the retina. We use models of these early visual pathways to transform natural images into statistical patterns of cortical input. The models include the receptive fields and non-linear response properties of the magnocellular (M) and parvocellular (P) pathways, with their ON and OFF pathway divisions. The results indicate that there are regularities in visual cortical input beyond those that have previously been appreciated from the direct analysis of natural images. In particular, several dark/bright asymmetries provide a potential account for recently discovered asymmetries in how the brain processes visual features, such as violations of classic energy-type models. On the basis of our analysis, we expect that the dark/bright dichotomy in natural images plays a key role in the generation of both cortical and perceptual asymmetries.

## Author Summary

Sensory systems must contend with a tremendous amount of diversity in the natural world. Gaining a detailed description of the natural world’s statistical regularities is a critical part of understanding how the nervous system is adapted to its environment. Here, we report that the well-established statistical distributions of basic visual features—such as visual contrast and spatial scale—diverge when separated into bright and dark components. Operations such as dark/bright segregation are key features of early visual pathways. By modeling these pathways, we demonstrate that the dark and bright visual patterns driving cortical networks are asymmetric across a number of visual features, producing previously unappreciated second-order regularities. The results provide a parsimonious account for recently discovered asymmetries in cortical activity.

## Introduction

One of the major insights of modern neuroscience is the recognition that regularities in the environment are embedded and exploited in neural circuitry [1, 2]. In the case of the visual system, this insight has led to the discovery of fundamental principles for encoding basic visual features, such as contrast, spatial scale, and edge orientation [3–5]. Environmental regularities also play a role in the higher level processes of visual perception and inference. For example, when hunting for berries, it is useful to have prior knowledge that berries tend to be small, round, and red. Perception relies on using such prior knowledge about the environment to make inferences from the imperfect visual signals [6–8]. It is thus clear that a detailed quantification of the statistical regularities in natural images is a critical part of understanding the visual brain. However, it is equally critical that these regularities be understood in the context of known pre-cortical visual transformations. Here, we describe an ensemble of robust statistical patterns in natural images that arise from the spatial layouts of bright and dark visual features. We furthermore show that these patterns, when combined with neural transforms in the early visual pathways, produce statistical regularities in the signals arriving to primary visual cortex. These regularities in the input to cortex provide a simple explanation for a range of recent neurophysiological findings: cells in visual cortex respond asymmetrically to brights and darks [9–17], with greater cortical responses to dark features particularly at high visual contrasts, low spatial frequencies, and far depths [12, 13, 15].

**Fig 1:**
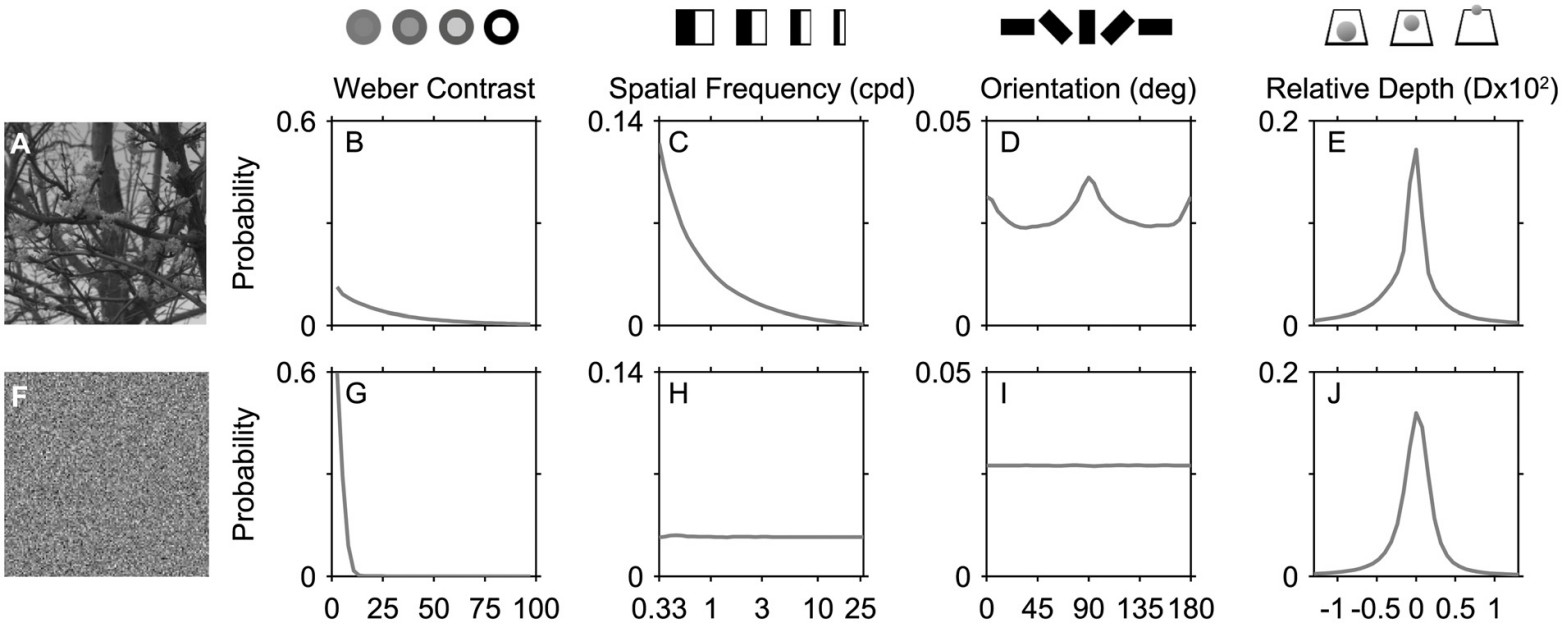
First-order statistical patterns in natural images. (A) Example of a natural image taken from a calibrated dataset [18]. The image has been gamma-corrected for visibility. (B-D) Probability density distributions for percent contrast (Weber), spatial frequency, and orientation calculated over an ensemble of 200 images. Contrast values for each pixel were calculated using calibrated image filter responses, and spatial frequency and orientation were calculated as magnitudes in the Fourier spectrum (See Methods for details). None of these distributions are uniform in natural scenes: low contrasts, low spatial frequencies, and cardinal orientations (0/180 = horizontal, 90 = vertical) are observed relatively more frequently than high contrasts, high spatial frequencies, and oblique orientations. (E) Probability density distribution for relative depth calculated over an ensemble of 31 depth maps from natural scenes [19]. Relative depth at each pixel was defined as the distance relative to the average distance of the local neighborhood. The most likely depth is near zero, with nearer depths (negative) and farther depths (positive) being relatively less likely. (F) Example of a white noise image with a Gaussian luminance distribution. (G-J) Using the same techniques as for natural images, the probability distributions were calculated over 25 noise images or noise depth maps. Distributions for spatial frequency and orientation are uniform for these images, whereas contrast and depth are both dominated by near-zero values. (Abbreviations: cycles per degree (cpd), degrees (deg), diopters (D)).

Fig 1 illustrates the known first-order statistical regularities of natural images for various basic visual features, derived here from a large calibrated image set [18, 19]. These features include visual contrast (Fig 1B), spatial frequency (or scale) (Fig 1C), edge orientation (Fig 1D), and relative depth (Fig 1E). Note that the ordinate scales differ between the different feature types. To understand how the structure particular to natural images contributes to these patterns, the same probability distributions are also shown for a set of randomly generated image pixels and randomly generated distances (Fig 1F–1J).

Natural images are dominated by low contrasts (Fig 1B) [5, 20, 21], but have relatively more high contrasts than the random pixels (Fig 1G). Natural images also contain more low spatial frequencies [22]—or large scale patterns—reflecting the fact that visual features tend to cluster together with other similar features (Fig 1C and 1H). In terms of edge orientation, natural images contain a slight bias towards having more cardinally oriented edges (Fig 1D and 1I) [8, 23]. This pattern can be attributed both to natural phenomena such as the horizon and tree lines, as well as to the carpentered lines of man-made structures. Finally, natural scenes can also be decomposed into a distribution of depths. In Fig 1E, we show the distributions of relative depths—distances compared to the average distance in the local neighborhood. This distribution is peaked near zero. A randomly generated set of distances resulted in a similar, although broader, distribution shape (Fig 1J). These first-order patterns in natural scenes have all been well-described in the previous literature. Here, we quantify a set of second-order patterns and show that these patterns arise naturally from interactions between first-order natural image properties.

The key to uncovering these regularities is a separate consideration of bright and dark visual features. In the early stages of visual processing in the retina, bright and dark features are processed separately via parallel pathways—one pathway encodes local areas of brightness (ON) and the other encodes local areas of darkness (OFF). This dark/bright dichotomy, however, has been largely overlooked in the study of natural scene statistics. There are three relevant observations that motivate our analysis: natural scenes contain more dark visual contrast [20, 24, 25], this dark bias increases at higher contrast levels [15], and dark visual contrasts also tend to be associated with farther relative depths [12, 19, 26]. These observations led us to hypothesize that the bright and dark visual features of natural images may differ along other dimensions as well. If this was the case, it would make sense for the visual system to exploit these differences.

Confirming and expanding on previous results, we found that bright and dark visual features are distributed asymmetrically in terms of their contrast levels and relative depths [12, 15, 26]. In addition, we found that the spatial frequency content of natural scenes differs substantially between brights and darks, with a higher dark bias at low spatial frequencies. We identify the origins of each of these regularities by synthesizing and analyzing noise images containing combinations of first-order image statistics. We then model the stages of early visual processing—which themselves contain several dark/bright asymmetries—and measure the statistical distribution of the cortical inputs from natural scenes after they have been processed through the ON and OFF pathways. Our analysis provides a parsimonious explanation for dark/bright asymmetries in well-known perceptual phenomena and recently discovered cortical phenomena.

## Methods

### Creation of Bright and Dark Images

**Fig 2:**
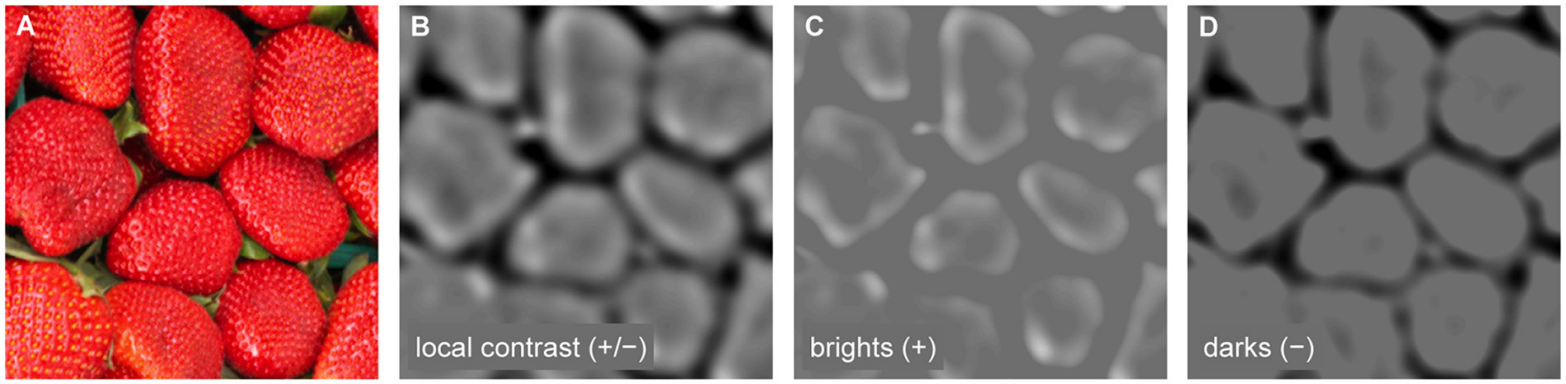
Separating images into bright and dark features. (A) Images from calibrated data sets [18, 19, 27] were filtered with normalized bandpass contrast operators—difference of Gaussians (DOGs). Filter outputs were divisively normalized by the local luminance as determined by a third Gaussian with a standard deviation equal to the larger Gaussian of the DOG. (B) The resulting images contained both negative and positive local contrast features. The colormap goes from black (negative contrast) to white (positive contrast), with middle gray indicating zero contrast. (C,D) These images were separated into brights (positive contrasts) and darks (negative contrasts).

We analyzed 200 images in the Van Hateren Dataset (IML format, 1536 × 1024 pixels) [18] for the main analysis and 80 images in the McGill Calibrated Color Image Database (TIF format, 768 × 576 pixels) [27] for an additional analysis. We converted pixel values to light intensity using the provided camera calibration information. The McGill images were additionally converted from color to grayscale by applying a standard conversion to the red (*r*), green (*g*) and blue (*b*) channels: *gray* = 0.299*r*+0.587*g*+0.114*b*. Based on the provided camera and image information, Van Hateren image pixels were assumed to be approximately 1 arcminute (arcmin) wide squares and McGill image pixels were assumed to be approximately half that size. To segment these images into their bright and dark features, we convolved them with 2D difference of Gaussian (DOG) filters (Fig 2A and 2B). Several different DOG sizes and shapes were used to ensure that any results were not idiosyncratic to a specific filter. For the main analysis, we report results for a DOG with a standard deviation for the central Gaussian (*σ*_*c*_) of 4 arcmin and a surround/center ratio (*σ*_*s*_/*σ*_*c*_) of 2. Results for the remaining DOG types are reported in the Supporting Information. These results include DOGs with smaller and larger central standard deviations (2 and 8 arcmin), and surround/center ratios (1.5 and 4). All Gaussians were unit sum, so the resulting filters were zero sum. We then applied a normalizing division to scale the filter response according to the local mean luminance. The responses were thus similar to percent contrast. The normalizing filter was equal in size to the surround Gaussian (*σ*_*n*_ = *σ*_*s*_). Thus, the resulting contrast filter response *c* for a pixel at location (*x*, *y*) was:

$$\begin{array}{c} c\left(x,y\right)=\frac{g\left(x,y;\sigma_{c}\right)-g\left(x,y;\sigma_{s}\right)}{g(x,y;\sigma_{n})}, \end{array}$$ (1)

where *g*(*x*, *y*;*σ*) is a 2D Gaussian of the form $\frac{1}{2}\pi \sigma^{2}\exp(-(x^{2}+y^{2})/2\sigma^{2})$. This contrast filter was based on previous work examining physiologically meaningful computations of contrast in natural images [20, 25, 28]. After convolution, the image edges were cropped by 1/2 filter width to remove boundary artifacts.

The range of values that result from convolving an image with *c* depends on the properties of the component Gaussians and therefore is not immediately comparable to the percent contrast values typically reported for experimental stimuli such as points of light or oriented gratings. So next, we converted these filter responses into units of equivalent contrast. As has been described previously [20], we applied the contrast filters to a range of individual images of spots of light or dark on a solid background. The diameter of the spot was always equal to the full width half maximum (FWHM) of the positive lobe of the DOG filter and the luminance values were uniform within the spot. Images were created with normalized luminance values ranging from zero to one, with the surrounding values always set to 0.5. The specific luminance values selected do not affect the calibration results. The percent contrast of these spots can be computed using two standard definitions: Weber contrast (*w*) and Michelson contrast (*m*). The equations for these two types of contrast were defined as follows:

$$\begin{array}{c} w=\frac{s-b}{b} \end{array}$$ (2)

$$\begin{array}{c} m=\frac{s-b}{s+b} \end{array}$$ (3)

where *s* is the luminance of the spot and *b* is the luminance of the background. Michelson contrast is typically used for gratings rather than spots, but we included this definition in our analysis for completeness. We created a lookup table for each filter and converted the filter response levels into the Weber or Michelson contrast of a single spot that would produce an equivalent response. We used linear interpolation for responses that fell in-between lookup table values.

This calculation results in lookup tables in which filter responses of equal and opposite magnitude will not necessarily translate to equal and opposite percent contrast values. These differences arise because the divisive term of the filter (*g*(*x*, *y*;*σ*_*n*_) in Eq 1) is affected by the luminance of both the central spot *s* and the background region *b* in a way that is not necessarily equivalent to the divisive terms of the contrast definitions. For Weber contrast, only the background luminance affects the divisive term, and for Michelson contrast, the spot and background contribute with equal weight (Eqs 2 and 3). In the case of the Weber definition, the filter response divisive term is relatively larger than the Weber divisive term when the spot is bright, and relatively smaller when the spot is dark. Thus, a bright spot tends to produce a lower filter response than a dark spot of the same Weber Contrast. In addition, the magnitude of these positive and negative contrast differences will scale with the size of the spot relative to the DOG filter, because more and more of the divisive Gaussian is affected by the spot. As stated above, we selected spots with diameters equal to the FWHM of the DOGs. This size produces a reasonable compromise between minimizing the positive and negative contrast differences, while still producing a robust filter response at ±100% contrast. Responses that fell outside of this range of equivalent contrasts were clamped to these maximum and minimum values—this was only 3.6% of responses in the main analysis. In the resulting values, positive contrasts indicate the locally bright visual points and negative contrasts indicate the locally dark visual points in the natural images. The images were segmented into their bright and dark features by taking either only the positive values (bright contrasts) or only the negative values (dark contrasts), in each case setting the remaining pixel values to zero (Fig 2C and 2D).

To make sure that our results were not idiosyncratic to this formulation of image contrast, we implemented an alternative contrast definition with only one free parameter and no need for equivalent contrast conversions. In this case, we simply low-pass filtered each image with a single 2D Gaussian and computed the Weber contrast (*w*) of each pixel, treating the original image pixel value as *s* and the low-passed local average value as *b*.

### Creation of Noise Images

We created five classes of noise images for comparison with natural images. Each class contained 25 distinct image/distance map pairs (1024 × 1024 pixels each). The first image class, *Gaussian white noise*, had a uniform frequency distribution and random-phase intensities drawn from a Gaussian distribution. Each subsequent image class was constrained to have an additional global characteristic typical of natural images. The next class, *Gaussian 1/*f*^*α*^ noise*, had a non-uniform spatial frequency distribution characterized by a 1/*f* ^*α*^ fall off (*α* = 1.3). The third class, *skew 1/*f*^*α*^ noise*, additionally contained intensity values drawn from a positively skewed distribution (the intensities were gamma-adjusted by raising each intensity value to a power of three). The fourth class, *skew 1/*f*^*α*^ oriented noise*, additionally contained boosted intensity values in orientation bands centered along vertical and horizontal orientations. The fifth class, *skew 1/*f*^*α*^ oriented noise with correlation*, was identical to the fourth class in the images, but contained modified distance maps.

Distance maps for all classes were also generated as Gaussian distributed values around a randomly selected average distance (mean distance = 40 meters, mean depth range = 80 meters), and attenuated high spatial frequencies (1/*f* ^*α*^ fall off with *α* = 1.3). For the fifth class of noise, the intensity values were scaled by a factor of 2.5 and subtracted from the distance values, imposing a modest negative intensity/depth correlation (mean *r* = -0.07). Noise images were separated into brights and darks and analyzed in the same way as the natural images.

### Distributions of Visual Contrast, Spatial Orientation, and Spatial Frequency

We computed contrast frequencies via a smoothed histogram of equivalent contrasts using equally spaced 5.4%-wide bins in steps of 2.7%. Values where contrast was equal to zero were excluded. To create spatial frequency and orientation distributions, we first computed the Fourier amplitude of each image, after multiplication with a circularly symmetric Hanning window. The amplitude spectrum was masked to the highest spatial frequency present at all orientations and to a low spatial frequency of 4 cycles per image. We then used 10°-wide, anti-aliased wedge masks to compute the mean amplitude centered around each orientation, in steps of 5°. We used 37 equally spaced log steps in spatial frequency and computed the mean across anti-aliased ring masks in cycles per degree (the width of each ring also increased logarithmically with spatial frequency). Each distribution was summed across all images and normalized to produce a probability density distribution for bright and dark contrast, orientation, and spatial frequency. Probability densities were normalized to the number of occurrences across both bright and dark contrasts in order to preserve the global dark/bright differences. In addition, the ratio of the summed distributions was calculated to produce the dark/bright amplitude ratio. Feature values with probability density of less than 10^−5^ were excluded from this ratio calculation. Because this analysis in the Fourier domain removed the DC offset (overall mean amplitude) of the images, we computed the overall amplitude difference separately and added it back in to dark/bright ratio distribution of orientations (for spatial frequencies, the mean difference is not plotted). For the distributions shown in the Introduction (Fig 1), the contrast probabilities were averaged over bright and dark points to get a one-sided contrast distribution, and all other analyses were performed prior to dark/bright segregation (i.e., on the original image pixel values).

### Distributions of Relative Depth

**Fig 3:**
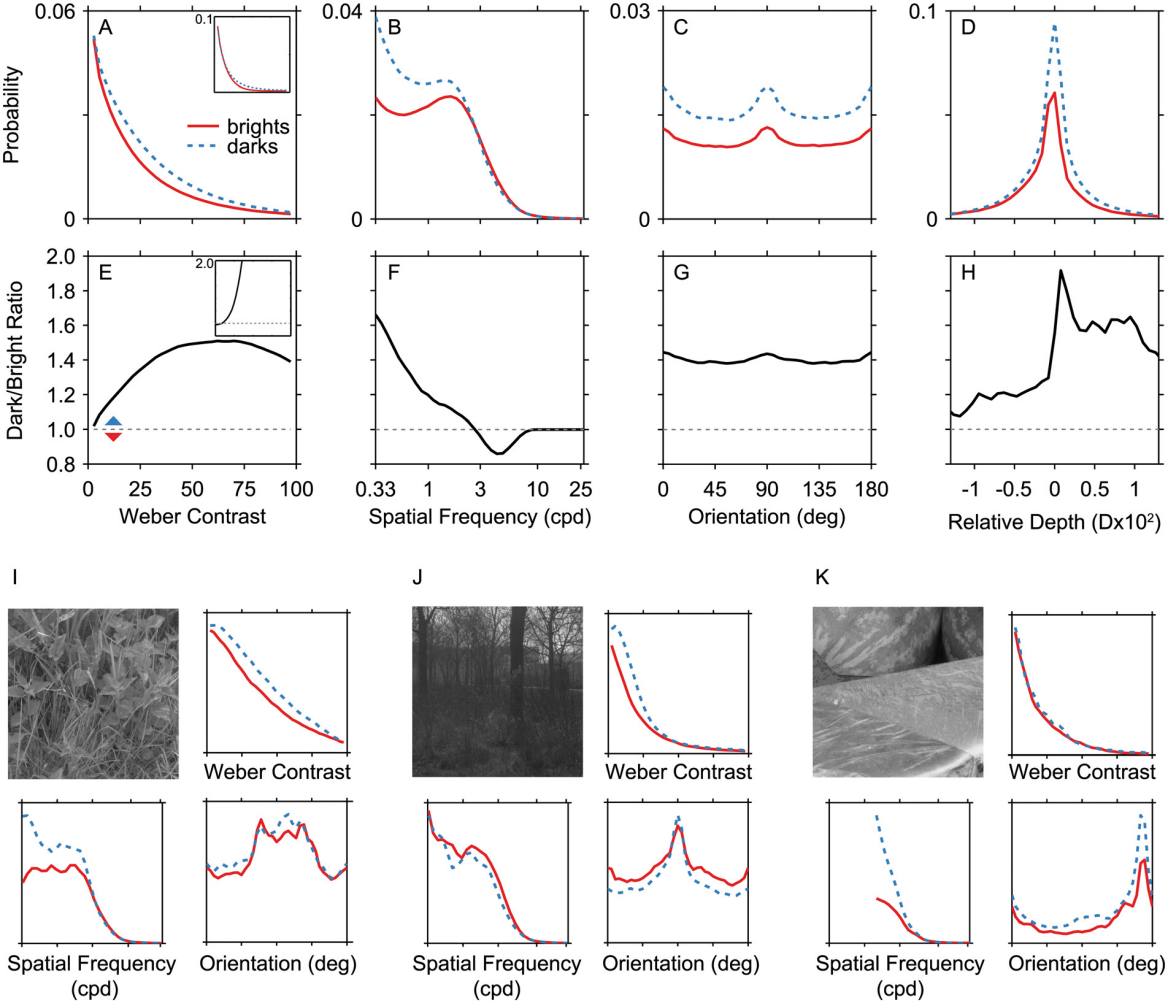
Dark and bright features are asymmetric in natural scenes. (A-D) Probability distributions are plotted as in Fig 1. Solid red lines show results for brights and dashed blue lines show results for darks. Results for contrast, orientation, and spatial frequency come from a single data set [18] and results for relative depth come from a second data set [19]. The inset in panel A shows the results if the Michelson definition of contrast is used instead of the Weber definition. Probability values are normalized across both dark and bright features. (E-H) For each value in the upper panels, the ratio of the dark probability to the bright probability is plotted. Values greater than 1 (dashed line, blue arrow) indicate that the feature is more likely to be observed as dark. The inset in panel E shows the results if the Michelson definition of contrast is used instead of the Weber definition. (I-K) Sets of results for three individual natural images are shown. Each group of 4 panels includes the original image, and normalized histograms for Weber contrast, spatial frequency, and orientation. The images have been gamma-corrected. Abscissa scales are the same as panels (A-D), and ordinates scales are normalized frequency within the single image (0-1). The spatial frequency data in panel K have a smaller range because this example comes from a second image set with smaller image sizes [27].

A separate dataset containing paired natural image and distance information (measured with a laser range scanner) was used to compute the relative depth distributions [19]. We used a subset of 31 images from this dataset that all had a pixel size of approximately 3 arcmin. We first converted the color images to grayscale using the same conversion described above. Next we converted the distance values to relative depth. The average local distance around each pixel was computed by convolving the distance map with a Gaussian filter with standard deviation of 30 arcmin. Distance maps had some missing or undefined values (for example, in the sky), so averages included only the valid distance estimates within the filter. Distance values were then converted from meters to Diopters (D; 1/meters) and the mean dioptric distance was subtracted out. This was done for two reasons: the binocular disparities encoded in early visual cortex scale linearly with diopters and are related to depth relative to a reference fixation. We computed the amplitude at each relative depth for bright and dark points by summing up the filter response amplitude in bins 1.6 × 10^−3^ D-wide in steps of 7.9 × 10^−4^ D. These distributions were highly kurtotic, so in Fig 3 the axes are clipped to contain 95% of the values. Again, for Fig 1 in the Introduction, an identical analysis was performed using the original pixel values.

### Simulation of Retinal Ganglion Cell Responses

We wanted to determine how the statistical patterns in natural images translate into statistical patterns of input to primary visual cortex. To do this, we simulated the receptive fields and response nonlinearities of earlier stages of visual processing, as these provide the relevant input to cortex. The normalized DOG contrast filters that we used to separate visual images into bright and dark features were modified to simulate the spatial receptive fields of retinal ganglion cells (RGCs) as reported in [29]. We modeled two classes of RGCs: a parvocellular pathway (P) comprised of midget cells and a magnocellular pathway (M) comprised of parasol cells. For each class, we also modeled receptive fields for foveal and peripheral cells and ON and OFF divisions. The standard deviations of the central Gaussians for each cell type in arcmin are given in Table 1. M receptive fields tend to be larger than P, peripheral receptive fields tend to be larger than foveal, and ON receptive fields tend to be larger than OFF. The values reported in [29] were collapsed across ON and OFF cell types, so to include the well-known tendency for ON cells of a given subclass to have larger receptive fields than OFF cells, we scaled the standard deviations by 110% to estimate the ON receptive field size and 90% to estimate the OFF receptive field size [30, 31]. To determine the surround Gaussian standard deviation, the central Gaussian’s standard deviation for each subclass was scaled by a factor of six [29]. All receptive fields were treated as zero sum prior to applying response nonlinearities, and were divisively normalized by a Gaussian equivalent to the surround region to simulate the effects of local light adaptation.

**Table 1:** Standard deviations for central Gaussians in model RGCs (in arcminutes).

Pathway (cell type)	Location	Center Std Dev (arcmin)	
		ON	OFF
Parvocellular (midget)	foveal	1.4	1.1
	peripheral	3.3	2.7
Magnocellular (parasol)	foveal	4.7	3.8
	peripheral	8.4	6.9

Model RGC receptive field sizes taken after [29]. Values are based on median *r*_*c*_ parameter reported in Table 1 of the previous report. For foveal regions, values were taken from the 0-5° range for P cells and the 0-10° range for M cells. Values from the cited Table are in terms of half width of a Gaussian fit at 1/*e* of the Gaussian’s maximum. These values were converted to standard deviation by dividing by $\sqrt{2}$.

RGCs have nonlinear and asymmetric contrast response functions. We modeled these response functions on previously reported direct measurements from the mammalian retina [31–33]. Both ON and OFF cell responses are rectifying, but the OFF response is more so. The ON RGCs begin increasing their spike rate when contrast is still negative, and thus have a higher response rate at zero and low contrasts [31, 32]. However, the ON response rate at high (near 100%) contrasts has been reported to be much lower than the OFF response at high (near -100%) contrasts [32, 33]. To model these contrast response properties, we first defined two functions that reflected the properties of the normalized ON and OFF cell responses as a function of stimulus Weber contrast. These were created by first taking a cumulative Gaussian function:

$$\begin{array}{c} f\left(w^{\prime };\mu_{f},\sigma_{f}\right)=\frac{1}{2}(1+\text{erf}(\frac{w^{\prime }-\mu_{f}}{\sigma_{f}\sqrt{2}})) \end{array}$$ (4)

where *w*′ is the equivalent Weber contrast of a filter response, erf(⋅) denotes the error function, and *μ*_*f*_ and *σ*_*f*_ were selected to reflect RGC response properties (37.5% and 30% for the ON responses, 60% and 20% for the OFF responses, respectively). The values of *f* were normalized to have a value of 1 at maximum contrast (100%). These functions were then modified to reflect the differences in preferred contrast polarity and response maximum between the two pathways:

$$\begin{array}{c} k\left(w^{\prime }\right)=\{\begin{array}{cc} \frac{1}{2}f\left(w^{\prime };37.5,30\right) & \text{if}\;ON\\ f(-w^{\prime };60,20) & \text{if}\;OFF, \end{array} \end{array}$$ (5)

where *k* is the expected RGC response. We then used these functions to remap the filter responses from equivalent Weber contrast into ON and OFF RGC response magnitudes. For example, a filter response reflecting positive Weber contrast of 25% would be mapped to a minor ON response (0.17) and an effectively zero OFF response. For -25% contrast, the OFF response would be present (0.04) and there would be a small ON response as well (0.01). This model assumes that the contrast response functions of RGCs are similar for different levels of mean luminance, although some recent work raises the possibility that mean luminance may interact with these responses [13]. For this analysis, filter responses with equivalent Weber contrast out of the range of modeled values (+/-100%) were clamped to this range. MATLAB code for simulating RGC responses with this model is provided in the Supporting Information (S1 File).

Finally, the simulated RGC response amplitudes for each subclass of cells were computed across all of the natural images. These were broken down into visual features as described in the previous Methods sections in order to estimate the expected distributions of cortical input magnitude over all of the visual features of interest.

## Results

### Dark and Bright Features Distribute Asymmetrically in Natural Images

The statistical properties of natural images differ along several dimensions between brights (Fig 3 solid red lines) and darks (Fig 3 dashed blue lines). The upper panels of Fig 3(A)–3(D) show the probability distributions of contrast, spatial frequency, orientation, and relative depth. The lower panels (3E–3H) show the ratio of dark to bright amplitude for each of these features, where values greater than 1 (dashed horizontal line) indicate a greater probability for darks. The overall bias towards dark features shown in these panels reflects the previously established dominance of darkness in natural scenes [20, 24, 25]. Across all visual features, the dark amplitude exceeded bright by a factor of 1.4. However, we can now see that this bias is not evenly distributed across the space of visual features.

Weber contrast has a steeper fall off for brights than for darks (Fig 3A). As visual contrast increases, the ratio of dark to bright increases as well (Fig 3E). As suggested by a previous analysis [15], this means that low contrast features are equally likely to be bright or dark, but relatively high contrast features are biased towards being dark. Dark Weber contrasts, however, are limited to be 100% in magnitude or below, whereas bright contrasts can go to infinity. Thus, very high contrasts (not shown) will be exclusively produced by brights. When the Michelson contrast definition is used instead, the results are qualitatively similar, but with a larger dark bias (see insets).

Spatial frequency has a shallower fall off for brights (Fig 3B and 3F). This manifests as a cross-over of the two curves. Note that the computation of bright and dark features is by its nature a bandpass calculation—features are determined to be bright or dark relative to the mean luminance of their local region. This bandpass filtering can be seen in the attenuation of low frequencies relative to the more typical frequency distribution shown in Fig 1C. Despite this bandpass effect, the probability for darks is still high at relatively low spatial frequencies, and exceeds that for brights. Interestingly, the relative probabilities of brights and darks reverse at higher spatial frequencies. At the highest frequencies present in the images, the probabilities become very similar. This occurs because the dark/bright image segmentation produces sharp edges at the transitions between brights and darks, which are identical in the two images. Orientation has a slight second-order asymmetry between brights and darks at cardinal orientations (Fig 3C and 3G), but is otherwise evenly distributed. Finally, relative depth (Fig 3D and 3H) exhibits a different pattern. There is a tendency for the dark bias to increase at farther depths (darks are on average 1.2 times more likely at near depths and this increases to 1.6 times more at far depths). Note that fewer images with both luminance and depth information were available, so the depth results are noisier than the results for the other features.

Examining the results for some individual images can suggest which properties of natural scenes give rise to these asymmetries. Fig 3I–3K show three example images with their individual frequency distributions for Weber contrast, spatial frequency, and orientation. The image in Panel I has feature distributions that are reflective of the average results across all of the images. Panel J shows an example image for which the dark bias at low spatial frequencies is absent, and Panel K shows an image for which the distributions of Weber contrast are similar for brights and darks. From these examples, we can hypothesize how the interplay between natural lighting, object surfaces, and shadows may lead to bright/dark asymmetries. In natural images, dark shadows tend to occur in the spaces between objects, whereas dark and bright textural features within objects may occur with similar frequency. This general pattern could lead to a dark bias at lower spatial frequencies (the spaces between objects), but no bias at high spatial frequencies (the details within objects), as seen in Panels I and K. In Panel J, the entire scene is extremely dark, and thus there is no clear distinction between objects and shadows. In the same vein, the prevalence of dark shadows and shading in natural scenes might tend to boost the presence of dark contrasts relative to bright contrasts. In the image in Panel K, there is only a single area of shadow, which might not be sufficient to accentuate this pattern. Similarly, it has been argued that shadows play a role in introducing a dark/far bias in natural images (not shown for these examples)[12, 19].

Distributions for a second set of natural images ([27]) are shown in S1 Fig. Additionally, we computed the same overall statistics using contrast filters of different sizes (S2 Fig), different shapes (S3 Fig), and different forms (Gaussians instead of DOGs; S4 Fig). Altering the contrast filter shape and dimensions effectively modifies the specific parameters used to determine whether a point in an image is locally bright or dark relative to the surroundings. The patterns shown in Fig 3 appear robustly in each of these additional calculations. The factor with the most noticeable effect on the scene statistics is the total size of the contrast filter, regardless of shape. Larger filters average over a larger area of the image in determining whether a point is bright or dark. These larger filters result in a shift of the low frequency dark bias towards lower and lower spatial frequencies, and accentuate the bright bias at high spatial frequencies. Applying these different contrast filters exposes the multi-scale way in which natural scenes differ in their bright and dark content. In order to understand which features of these dark/bright differences are relevant to the visual system of the brain, however, it is essential to create physiologically-based contrast filters, which we will describe in the Results section on modeling the early visual pathways.

### Dark/Bright Asymmetries Arise from Statistical Regularities in Natural Images

**Fig 4:**
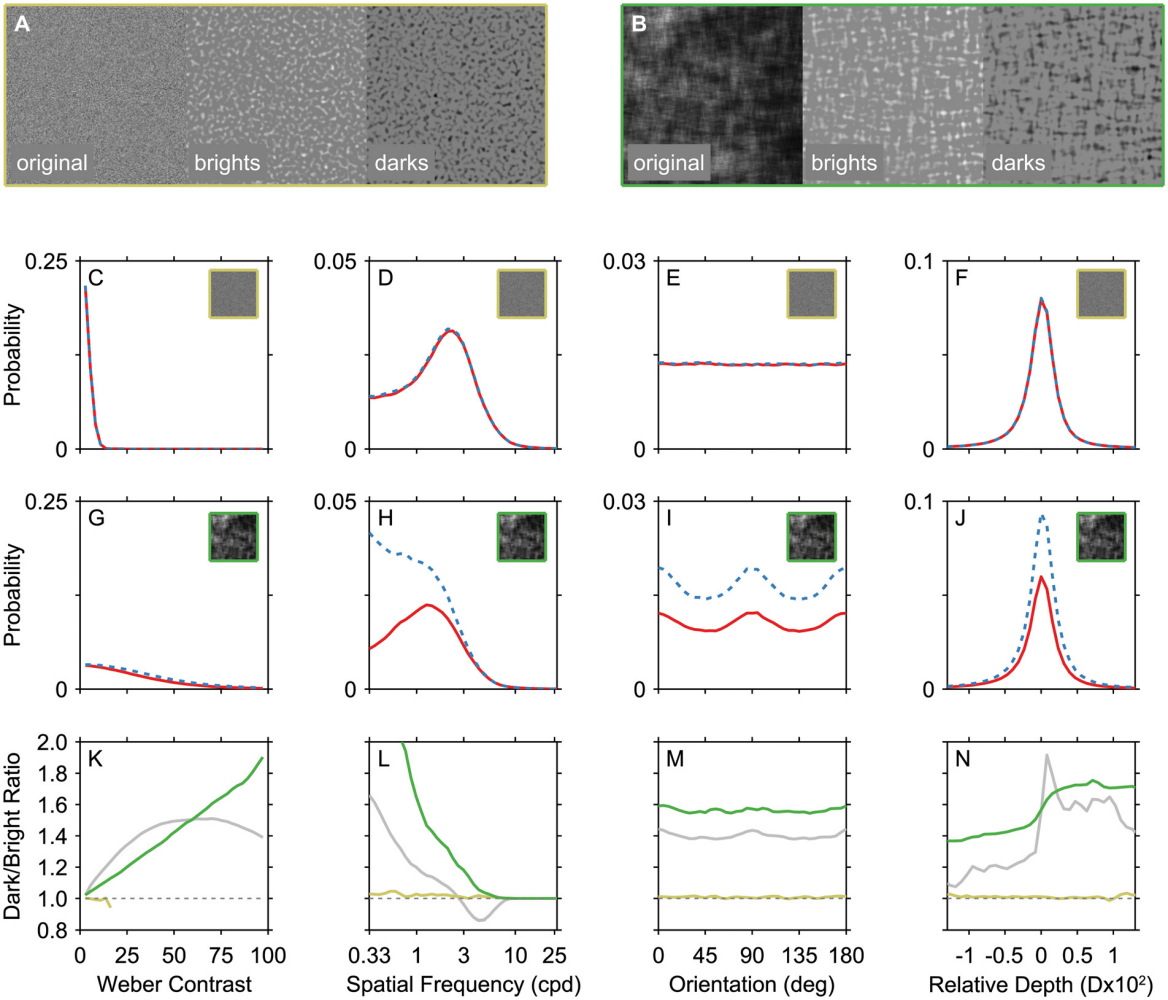
Dark/bright asymmetries arise from global statistical image properties. (A,B) White noise images (indicated throughout with yellow outlines) and naturalistic noise images (indicated throughout with green outlines) were separated into dark and bright features as described for the natural images analysis. (C-F) In white noise images, the distributions of visual features are identical for brights and darks. (Note that the spatial frequency distribution for these images is not flat due to the bandpass nature of the contrast filters.) (G-J) Naturalistic noise images were generated to reflect several global features of natural images, but were otherwise unstructured. In naturalistic noise images, many of the dark/bright asymmetries in natural images are reproduced. (K-N) Dark-to-bright ratios are shown for each type of noise as in Fig 3 to further illustrate the areas of agreement and disagreement. Yellow lines indicate white noise, green lines indicate naturalistic noise, and grey lines show the results for natural images from Fig 3 for comparison.

We wanted to understand the underlying source of the dark/bright asymmetries in natural images. Are they due to the specific geometric and lighting patterns in natural scenes, or could simpler statistical patterns account for these biases? To answer this question, we performed identical analyses on synthetic noise images: white noise with a Gaussian luminance distribution (Fig 4A) and structured noise that we will call *naturalistic noise* (Fig 4B). Naturalistic noise contains four first-order patterns from natural scenes: a positively skewed luminance histogram (more dark points than bright points) [34], a fall off in spatial frequency (*f*) amplitude determined by the function 1/*f* ^*α*^, a predominance of vertical and horizontal orientations, and a negative correlation between the intensity of a pixel and the pixel distance.

Thus, naturalistic noise contains common patterns in the amplitude spectrum of natural images, but lacks the phase characteristics that result from recognizable image features, such as object boundaries, shadows, and occlusions. Do either of these types of noise images contain any of the dark/bright asymmetries found in natural images? If white noise images contain asymmetries, it would suggest that the asymmetries are due to an inherent bias present in the current definition of brights and darks, rather than a systematic pattern particular to natural images. If naturalistic noise images contain asymmetries, it would suggest that the basic first-order patterns of natural images are sufficient to drive these asymmetries, independent to particular geometric or lighting features. If geometric features are necessary for producing dark/bright asymmetries, however, then naturalistic noise should fail to reproduce the dark/bright asymmetries from natural scenes. Thus, to the extent that naturalistic noise includes dark/bright asymmetries absent in white noise, we can attribute these effects to one the four first-order patterns that were imposed on these images.

The lower panels of Fig 4 show the probability distributions for these two types of noise. Panels C-F are the results for white noise, and panels G-J are the results for naturalistic noise. White noise images clearly do not contain the same dark/bright biases found in natural scenes. However, the simple model of global image patterns in naturalistic noise closely reproduces many of these biases in detail. This is further illustrated in panels K-N, which show the same dark-to-bright ratios as plotted in Fig 3 (yellow lines: white noise, green lines: naturalistic noise, gray lines: natural scenes). Several of the biases from natural scenes are qualitatively present in the naturalistic noise.

By deconstructing the four types of structure that were imposed on naturalistic noise, it is possible to hypothesize about the causes of the dark/bright asymmetries in natural images. (See Supporting Information S5 Fig for results from the intermediate patterns of noise that support these conclusions.) First, a positively skewed histogram increases the prevalence of dark image regions and accentuates greater dark contrasts (Fig 4K). Note that the white noise images are so dominated by low contrasts that almost all are below 16% (for visibility, the contrast of the example white noise bright and dark images in panel A have been increased by a factor for 3 relative to the naturalistic noise).

On top of this, a fall off at high spatial frequencies leads to images in which larger dark regions are clustered together separately from bright regions. That is to say, neighboring pixel intensity values become spatially correlated. Recall that the definition of local contrast entailed a normalization stage. This normalization stage converts the luminance differences into percent luminance difference, similar to the effect of local light adaptation in the early visual system [35]. Given the clustering pattern of naturalistic noise, it makes sense that normalized local contrast is boosted at the relatively low spatial scales at which dark clusters emerge (Fig 4L). This is because the contrast boosting within dark pixel clusters will only occur for spatial scales at which the normalization area of the contrast filter can fall mostly or entirely within a cluster of dark pixels. These dark clusters in naturalistic noise may be serving a similar function to the attached and unattached shadows if objects in natural scenes. This analysis suggests that two key factors contribute to boosting dark low spatial frequencies: local light adaptation and a 1/*f* ^*α*^ spatial frequency distributions. Given that each of these factors are common in natural vision and images, we can predict that the dark/bright asymmetry in spatial frequency may be a nearly universal pattern for most biological visual systems. Note that simply generating 1/*f* ^*α*^ noise with Gaussian luminance distributions is sufficient to produce images with this bias, without including the other features of naturalistic noise (S5 Fig). Finally, having an overall cardinal orientation bias produces largely symmetric distributions for brights and darks (Fig 4M) and adding a slight negative intensity/depth correlation (as has been observed in natural scenes [19]) reproduces a near/far asymmetry (Fig 4N).

This analysis shows that dark/bright asymmetries can arise from very simple statistical regularities that are shared by natural images, but are not specific to them. We propose that these regularities are likely a pervasive property of the input received by the visual system. However, just because naturalistic noise can reproduce these patterns does not prove that the structural properties that we imposed on these noise images are the actual or exclusive sources of the biases in natural images. For example, natural images contain edges and sharp object boundaries that are absent from all of the examined noise images. It is very likely that this spatial phase property of natural images contributes to their dark/bright asymmetries, because object edges are often the source of both luminance and depth discontinuities [36].

### Early Visual Pathways Carry Different Image Statistics Forward into Visual Cortex

Cells in primary visual cortex respond asymmetrically to the presentation of bright and dark visual features. The most striking asymmetry is a general dominance of cortical cells and cell activity devoted to processing darks. This *dark dominance* has been reported in multiple species, including cat, tree shrew, and human and non-human primates [9–17, 37]. Within this general dominance, a few additional patterns have started to emerge. The results of three studies show a tendency for this dark dominance to increase with greater visual contrast [12, 13, 15]. Two studies also found a greater dark dominance for lower spatial frequencies [13] and far depths (measured via cell tuning for the binocular disparity between the two eyes)[12].

**Fig 5:**
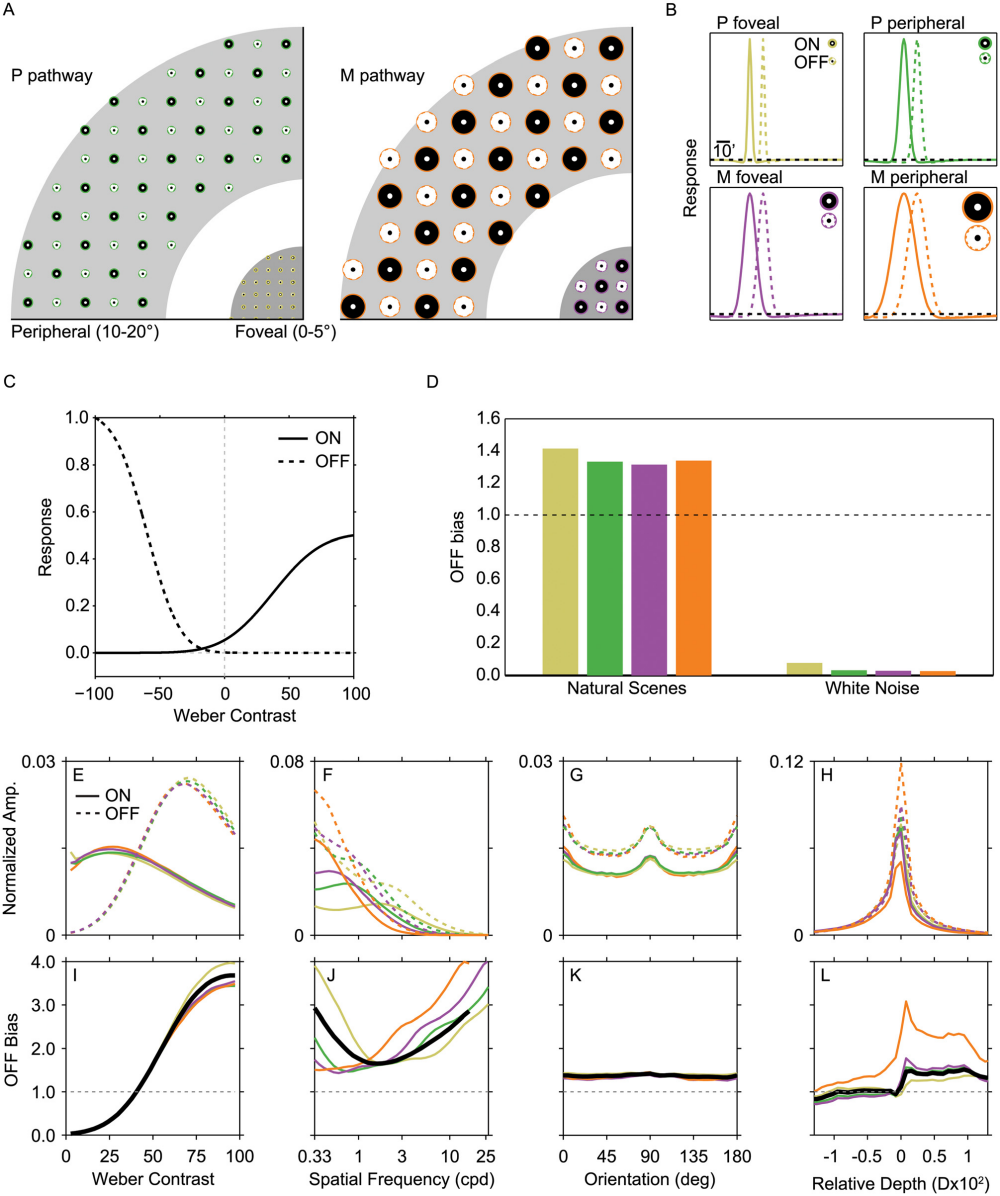
Patterns of dark dominance in cortical input. (A) Two schematics of retinal location illustrate the layout of 8 RGC subpopulations: P pathway and M pathway, foveal and peripheral, ON and OFF. ON (bright center, dark surround) and OFF (dark center, bright surround) cells are illustrated as alternating for clarity, however each subpopulation actually fully tiles the retinal space. Bright values indicate excitatory regions and dark values indicate suppressive regions. P cells are smaller and more numerous than M cells, and foveal cells of both types are smaller than peripheral cells. Four colors are used throughout to indicate each subpopulation: P foveal (yellow), P peripheral (green), M foveal (purple), and M peripheral (orange). (B) Illustrations of the spatial receptive fields of the simulated retinal ganglion cells. Each of the four plots shows the receptive fields for ON and OFF cells of one subpopulation. Each line shows a middle slice through the isotropic 2D DOGs used to simulate RGC receptive fields. Solid lines show the extent of ON receptive fields and dashed lines show the extent of OFF receptive fields, offset laterally for visualization. The black dashed line indicates zero response. OFF receptive fields respond positively when the center is darker than the surround and visa versa. Because the surrounding Gaussian has a large standard deviation, the suppressive surrounds appear very weak in these plots. Icons located within each plot show the ratio of center-to-surround standard deviations. Details of the receptive field parameters can be found in the Methods. (C) Models of the contrast response nonlinearities previously measured for retinal ganglion cells. (D) The overall ratio of OFF to ON cortical input from each pathway for natural images and white noise images. The horizontal dashed line at 1 indicates equal OFF and ON input, values greater than 1 indicate an OFF bias. (E-H) Normalized amplitude (Amp.) distributions for each visual feature are shown for each subpopulation. For Weber contrast, ON and OFF responses were only aggregated for positive contrasts and negative contrasts, respectively. (I-L) The OFF bias was computed as the ratio of the summed OFF responses to the ON responses over all input images.

We showed that natural images have more dark features overall, and particularly at high contrasts, low spatial frequencies, and far depths (Fig 3E–3H). Could cortical dark dominance reflect an adaptation to these patterns in the incoming visual signals? One previous study showed good agreement between the pattern of dark dominance in primary visual cortex and the distribution of contrasts in natural scenes [15]. However, it is well-known that the pre-cortical stages of visual processing contain substantial asymmetries in their treatment of brights and darks, so it is not possible to draw conclusions about cortical input patterns from the properties of natural scenes alone. For example, the responses of ON RGCs are greater at low contrasts than OFF cells [31, 32]. This difference could easily tip the balance away from dark dominance in the afferent signal to visual cortex. We wanted to determine how pre-cortical processing asymmetries would affect the subsequent input patterns to visual cortex. To do this, we simulated the operations of the receptive fields and nonlinearities of eight RGC subpopulations and applied them to natural images. The receptive field shapes for each subpopulation and the contrast response nonlinearities for the ON and OFF divisions are shown in Fig 5A–5C. We treat the ratio of OFF-signal to ON-signal (*OFF bias*) as a prediction of the ratio of cortical input received for dark and bright visual features over typical visual experience.

For each subpopulation, the OFF bias over a set of natural scenes tended to be greater than 1 (Fig 5D). We wondered how much of this OFF bias was inherent to the RGC responses, so we also performed the simulation on a set of white noise images. As expected, the OFF bias shrank to less than one for this image content. This is because white noise images are dominated by low contrasts (Fig 4C) and the ON RGC response is greater than the OFF response at low contrasts (Fig 5C). These global OFF/ON ratios are affected not just by the RGC response properties, but also by the way local contrast is defined. Recall that the calculation of contrast in these images required the selection of a calibration spot stimulus. Pilot testing indicated that the global OFF bias was sensitive to this spot size, because changing the size creates shifts in the resulting contrast histograms of natural and synthetic images. Thus, the predicted OFF bias could take on a range of values, and in some scenarios reversed to be an ON bias. It remains an open question exactly how to relate RGC responses measured in the laboratory (which we used to create this model) to their responses to the complex contrast patterns in natural scenes (which we are trying to infer). Importantly, the non-uniformities in the OFF bias across visual features, discussed below, were largely robust to the selection of spot size. These second-order patterns thus provide a potential avenue for investigating the encoding on bright and dark features independent of a specific contrast model.

Each subpopulation also has its own signature feature distribution (Fig 5E–5H). Features are plotted as normalized amplitude: the predicted amount of that subpopulation’s overall signal devoted to that feature. This is determined by both the scene properties and the cell responses. For example, because ON RGCs respond above baseline to low contrast features, the amplitude for all ON subpopulations is relatively high at low contrasts, but lower at high contrasts because high contrasts are overall less likely to occur (Fig 5E). In comparison, all OFF RGCs have a low amplitude at low contrasts and begin increasing their amplitude as contrast increases. Additionally, the larger receptive fields associated with the M pathway and the peripheral retina produce less signal attenuation at low spatial frequencies (Fig 5F). When plotted in terms of OFF bias for each subpopulation (Fig 5I–5L), it becomes evident that the smallest receptive fields produce the largest OFF bias at low frequencies, as predicted by the natural images analysis (Fig 5J). The asymmetric receptive field sizes for ON and OFF (ON larger than OFF) lead to a second boost of OFF input at higher spatial frequencies. These frequencies are much higher than have currently been measured in primary visual cortex. For example, Kremkow et al. ([13]) described a dark bias increasing from higher to lower spatial frequencies in the range of 0.03–0.75 cpd, a range over which the P pathway RGC models clearly show the same pattern, but did not report results for higher frequencies. Conversely, the near/far bias is strongest in the M pathway (Fig 5L). The black lines in Fig 5I–5L show a weighted average response assuming that the P pathway cells are nine times more numerous than the M pathway cells [29]. It is clear from these averages that this simulation predicts more afferent signals for dark features overall, and particularly at higher contrasts, low spatial frequencies (and very high ones) and far depths.

Thus, specific patterns of cortical dark dominance [12, 13, 15] may be matched to the input from afferent pathways to primary visual cortex. In addition to these major patterns, a previous study found that dark dominance does not vary substantially with spatial orientation, which is also consistent with the modeling results (Fig 5G and 5K)[13]. Finally, two previous studies reported a reversal towards bright dominance at low contrasts [13, 15]. This pattern is not present when contrast distributions are measured from natural images directly (Fig 3A), but emerges in the modeling due to the different ON and OFF response nonlinearities (Fig 5I). The model predicts additional bias patterns, such as the dipper shape as a function of spatial frequency and the M and P pathway differences, that can be tested experimentally.

## Discussion

### Implications for Classic Energy Models

According to hierarchical visual processing models, cortical receptive fields for basic visual features such as spatial orientation, spatial frequency, motion, and binocular disparity arise from a confluence of the ON and OFF pathways [38]. A hallmark of the energy models that have classically been used to describe these cortical receptive fields is the symmetric combination of opposite contrast polarity input [39–41]. For example, a complex cell might increase its firing rate when a vertically oriented edge is visible regardless of whether the edge is bright or dark. This could be achieved by receiving equal input from a pair of simple cells that each has a receptive field oriented to respond to either a dark (OFF) or bright (ON) vertical edge. Contrast invariance has been considered an advantage of complex cells, because they become pure detectors of the target visual feature and discard irrelevant information. However, responses from recent recordings of visual cells violate this pure contrast invariance assumption of energy models [10, 11, 14, 42]. One outcome of the current work is to suggest a functional explanation for this discrepancy.

**Fig 6:**
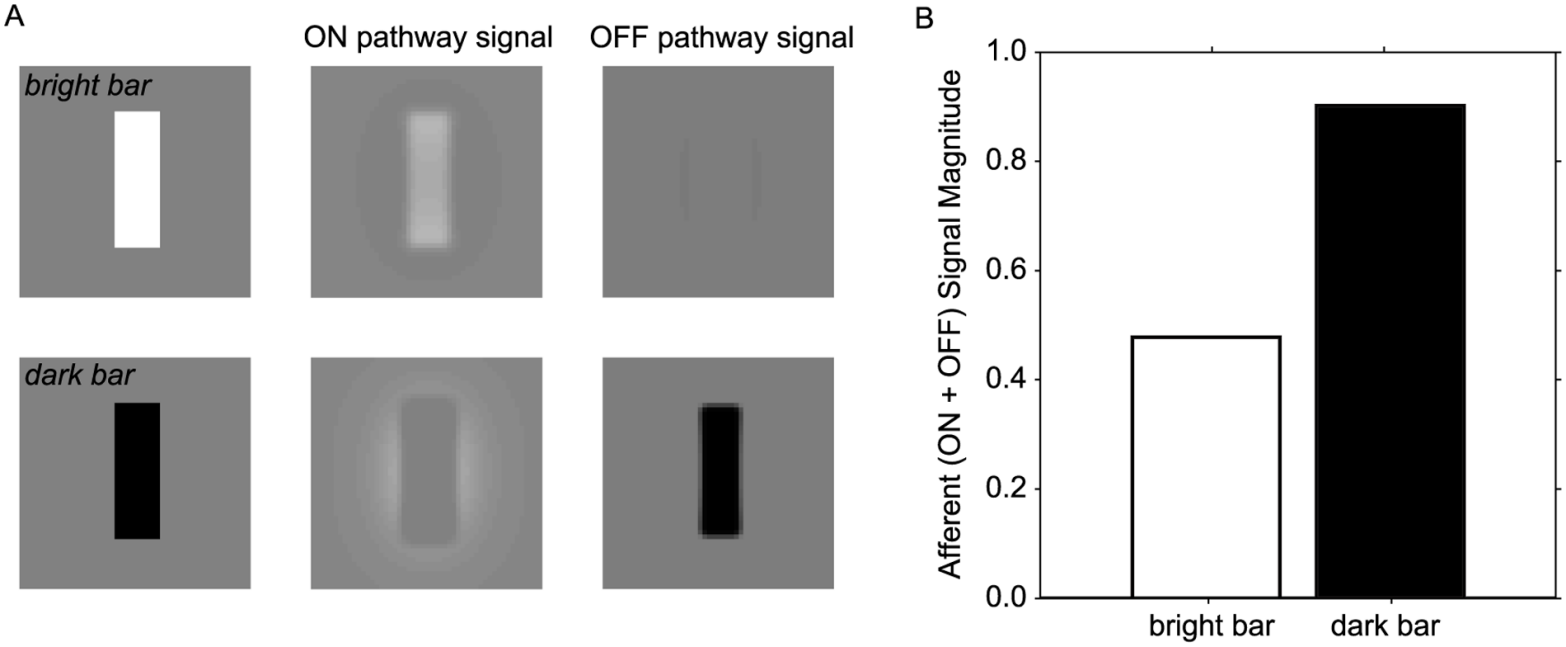
Patterns of dark dominance in cortical input from simple visual stimuli. (A) Images of a small bright bar and dark bar (10 arcmin wide, 30 arcmin tall, on a 50 arcmin square) were shown to the model RGCs. For each bar, the response for all RGCs over the whole square, (both ON and OFF) were summed together and weighted by a factor of 9:1 for P pathway to M pathway. Example responses are shown for the foveal P pathway cells. (B) Resulting prediction for the magnitude of the afferent signals to primary visual cortex stimulated by the bright and dark bars.

We propose that two factors could underly these energy model violations. The first is simply the instantaneous effect of the early visual nonlinearities on the afferent visual signal. The second is a cortical process of long-term potentiation and depression over visual experience—connections that are more active are potentiated (or up-weighted) and those that are less active are depressed (or down-weighted). To examine the first factor, we asked if two stimuli of equal and opposite Weber contrast (such as those used in physiological experiments) might generate afferent visual signals of unequal magnitude. Based on our analysis, we predicted that negative Weber contrast should produce a larger afferent signal than an equal positive contrast. We illustrate this in Fig 6. Images of small vertical bars with 100% positive or negative Weber contrast were presented to our RGC models (Fig 6A). The resulting ON and OFF pathway signals are illustrated in the right panels, with bright values indicating the presence of an ON response and dark values indicating the presence of an OFF response. These panels only show the responses for P pathway foveal cells. We summed ON and OFF signals across all pathways over a small region containing the bar to simulate the overall afferent activity reaching visual cortex. The simulation activity for the dark bar was 1.9 times greater than for the bright bar (Fig 6B). This calculation just provides a single example of this ratio, because the exact ratio varies depending on the size of the bar, the image area over which the responses are pooled, and the retinal location being modeled (here we included both foveal and peripheral results to get an average prediction). Nonetheless, if visual complex cells instantiating an energy-model-type computation responded with the same response gain to ON and OFF pathway signals generated by a stimulus such as this one, we would still predict a greater response to dark stimuli based on the early visual nonlinearities alone. Note, however, that some studies have reported lower dark biases in LGN cells and cortical input layers, suggesting a lower input OFF bias that is not consistent with this example [10, 13].

Previous studies have reported the OFF bias in populations of V1 neurons as being on average ∼ 1.2–3 (in cat and monkey, depending on the cortical layer [10, 12, 13]). Similar OFF biases have been reported in local field potential (LFP) and electroencephalogram (EEG) recordings in monkeys and humans [13, 37]. Generally, models of synaptic dynamics predict that a neuronal circuit that starts out with equal or arbitrary synaptic weights will drift towards an equilibrium state in which the weighting value for a given synapse is roughly proportionate to the activity level of the presynaptic neuron [43, 44]. The more active synapse will be up-weighted and the less active synapse will be down-weighted. Our results are consistent with the proposal that the dark bias in afferent signals is inherited and may also be amplified in visual cortex [10, 11, 13]. Indeed, prior work has demonstrated that this OFF bias reflects both a decrease in ON responses and an increase in OFF responses from V1 input layers to output layers, as predicted by combined potentiation and depression [11].

As described in the previous section, the contrast, scale, and depth dependent patterns in natural scenes also qualitatively agree with recent physiological measurements [12, 13, 15]. However, additional work is needed to establish the level of quantitative agreement on a feature-by-feature basis.

### Implications for Visual Perception

Taken together, our results and those of previous studies suggest that the cortical asymmetries in encoding dark and bright visual features reflect a highly specific match to the visual input coming from the natural environment. But for these asymmetries to be adaptive, they must also confer a performance advantage on the organism.

In many cases, visual perceptual performance tends to be enhanced for dark patterns relative to brights. This enhancement has been demonstrated for contrast sensitivity ([45–48]), speed and accuracy of target detection ([16, 49]), judgments of texture variance ([50]), and several other tasks (see [51] for review). It should also be noted that several of the same studies and others have identified conditions under which perception of brights and darks appear to be highly similar ([16, 45, 46, 49, 52, 53]). It is nonetheless appealing to think that the cortical asymmetries described here may be the underlying substrate of a “dark advantage” in some perceptual tasks. By allocating greater processing resources for dark features, the visual system is in effect making a prior assumption that certain visual features are more likely to appear as darks than to appear as brights.

**Fig 7:**
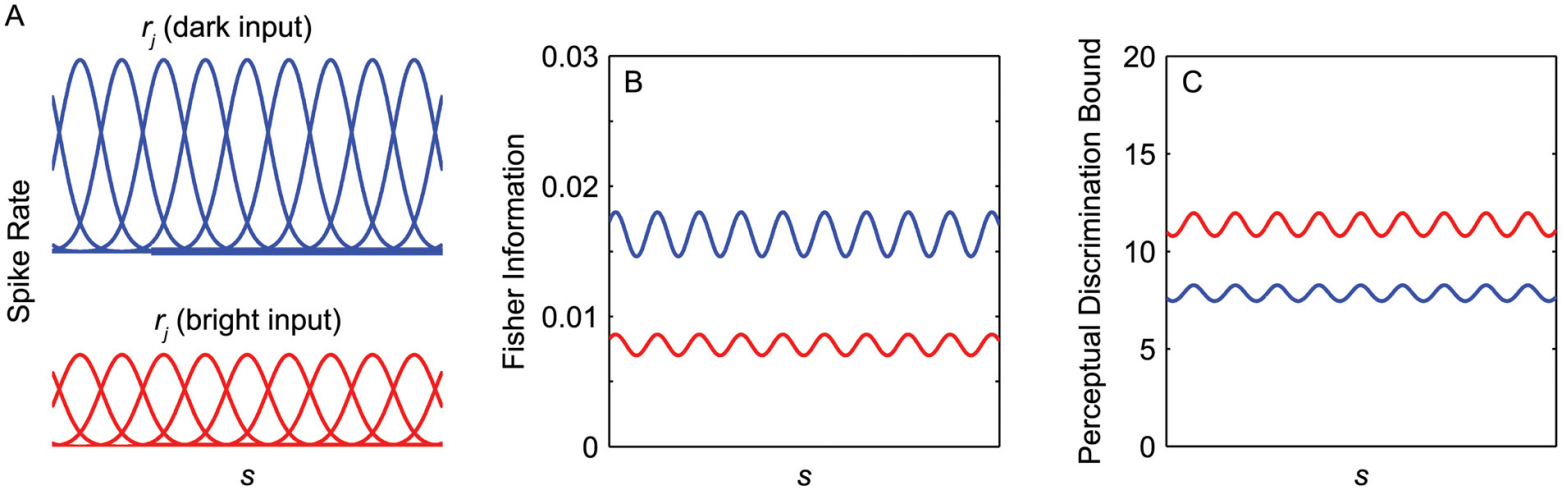
A population model can be used to predict biases in perceptual discrimination. (A) Illustrations of complex cell tuning functions for an imaginary visual feature *s*. Each cell’s tuning function is illustrated as a Gaussian function. We model the population as a set of identical functions *r*_*j*_ that are uniformly spaced over the range of stimulus values. The responses are shown separately for dark features (blue lines) and bright features (red lines). To simulate the dark bias in primary visual cortex, we model the dark-input responses as being 2 times greater than bright-input responses. (B,C) We computed the Fisher Information and lower perceptual discrimination bounds of the population responses to brights and darks assuming a maximum spike rate of 25 spikes per second in response to dark input.

We quantified this prediction using an information-theoretic approach and a neuronal population model that is illustrated schematically in Fig 7A. We start by considering a population of complex cells that are all tuned for a particular visual feature. The population is parameterized as a family of Gaussian tuning curves that uniformly tile the space of a scalar visual feature *s*. The shape of the tuning function for the *j* ^*th*^ neuron in the population is determined by:

$$\begin{array}{c} h_{j}\left(s\right)=e^{\frac{-{\left(s-\mu_{j}\right)}^{2}}{2\sigma_{j}^{2}}}, \end{array}$$ (6)

where *μ*_*j*_ is the value of *s* for which the response of neuron *j* is at its peak, and the standard deviation *σ*_*j*_ is the same for all neurons. The function values range from 0-1, with a value of 1 for the preferred stimulus. The absolute spike rate of a complex cell to a given stimulus (*r*_*j*_(*s*)) is determined by scaling this tuning shape by the maximum spike rate of the cell (*R*):

$$\begin{array}{c} r_{j}\left(s\right)=Rh_{j}\left(s\right). \end{array}$$ (7)

Consistent with a wide range of physiological studies, we assumed that the overall spike rates of the cells will be two times greater when the visual feature is presented with dark contrast (blue lines in Fig 7A) than when it is presented with bright contrast (red lines Fig 7A) [10, 12, 13, 37], but that the tuning curves will otherwise be similar in shape [12, 13].

The expected information value of the population activity at each value of *s* can be quantified as the Fisher information. This Fisher information can be approximated as:

$$\begin{array}{c} F\left(s\right)\approx \sum_{j=1}^{J}\frac{{\left(r_{j}^{\prime }\left(s\right)\right)}^{2}}{r_{j}\left(s\right)} \end{array}$$ (8)

where *J* is the total number of complex cells in the population, and $r_{j}^{\prime }$ is the first derivative of the response curve of the *j* ^*th*^ neuron with respect to *s* [54, 55]. Intuitively, the Fisher information of a population increases when tuning curves are steeper and/or more densely packed. This information measure is plotted in Fig 7B for dark and bright features. Because the increased response gain for dark features makes the tuning curves steeper, these responses have a higher level of Fisher information.

We can show that the ratio of the Fisher information in the dark and bright responses is equal to the ratio of the maximum response rates for dark and bright stimuli. First, substituting *Rh*_*j*_(*s*) for *r*_*j*_(*s*) in Eq (8) yields:

$$\begin{array}{c} F\left(s\right)\approx \sum_{j=1}^{J}\frac{{\left(Rh_{j}^{\prime }\left(s\right)\right)}^{2}}{Rh_{j}\left(s\right)}. \end{array}$$ (9)

Since *R* is a constant, this equation simplifies to:

$$\begin{array}{c} F\left(s\right)\approx R\sum_{j=1}^{J}\frac{{\left(h_{j}^{\prime }\left(s\right)\right)}^{2}}{h_{j}\left(s\right)}. \end{array}$$ (10)

Because we are calculating Fisher information for the same population (just with bright or dark input stimuli), the sum of the tuning curves drop out in the ratio of Fisher information between dark and bright input. This leaves:

$$\begin{array}{c} \frac{F_{d}\left(s\right)}{F_{b}\left(s\right)}=\frac{R_{d}}{R_{b}}, \end{array}$$ (11)

where subscripts *b* and *d* indicate the Fisher information and mean firing rates for bright and dark input, respectively.

Turning to the perceptual implications of the model, it has been shown that the lower bound on perceptual discrimination can be predicted from the Fisher information in the cell population. This lower limit is simply:

$$\begin{array}{c} \delta \left(s\right)\geq \frac{\Delta }{\sqrt{F(s)}} \end{array}$$ (12)

where Δ is a constant that is determined by the experimental paradigm [56]. This lower bound is shown in Fig 7C. Assuming that the experimental paradigm is the same for assessing discrimination thresholds for brights and darks, we can now calculate the predicted dark advantage. We will define the dark advantage as the ratio of the discrimination thresholds for bright and dark stimuli:

$$\begin{array}{c} \frac{\delta_{b}\left(s\right)}{\delta_{d}\left(s\right)}=\sqrt{\frac{F_{d}\left(s\right)}{F_{b}\left(s\right)}}=\sqrt{\frac{R_{d}}{R_{b}}}. \end{array}$$ (13)

So, given the assumption that the dark and bright responses are identical up to a spike rate scale factor, it is simple to calculate that if *R* is approximately two times higher for darks, *δ*_*b*_(*s*)/*δ*_*d*_(*s*) will be $\sqrt{2}\approx 1.4$, indicating a 40% lower discrimination threshold. This value is in keeping with a recent extensive study of the perceptual dark advantage at supra threshold contrasts (including eleven different experiments), which found that it ranges from 19% to 43% over a variety of perceptual tasks [51]. This agreement does not hold for contrast discrimination, which had a substantially higher dark advantage than the other tasks. However, the current analysis applies to neurons with Gaussian tuning profiles, which likely does not reflect the manner in which contrast is encoded in the early visual system.

Here, we have used a simplified case in which the cortical dark bias is the same for all values of *s* and the neuronal population is uniform. More work will be needed to determine if this cortical dark bias and perceptual advantage are distributed across visual features in a way that agrees with the more complex natural scene patterns reported in our results. A clear prediction of this model is that the relative dark advantage for two values of a particular feature should have the same sign as the relative OFF bias (Fig 5J–5L). Because this OFF bias in the environment varies across visual features, these variations may provide an explanation for why some experimental paradigms reveal a dark bias and others do not. For example, one might predict that the perceptual dark bias would be much smaller for stimuli with mid-range spatial frequencies (1–4 cpd) relative to higher or lower frequencies.

### Future Directions

One challenge to determining the statistics of cortical input is developing a more detailed model of the early visual pathways, particularly when it comes to the simulation of contrast response functions. Our ability to predict cortical input statistics will be improved as we learn more about how pre-cortical cell response properties are affected by the spatial patterns of natural input. For example, recent work showed that the difference in the ON and OFF RGC receptive field sizes—and perhaps their different response functions as well—fluctuate based on the mean luminance of a stimulus [13]. Factors such as these will clearly interact with the complex visual input patterns from natural scenes in ways that are difficult to predict without a more complete description of RGC responses to a wide variety of stimuli.

Another interesting avenue for future work would be to examine how visual statistics might vary as a function of retinal eccentricity. For example, observers may tend to preferentially fixate the detailed, high contrast areas of a visual scene. Thus, neurons representing foveal and peripheral regions may be tasked with encoding different distributions of contrast and spatial frequency. Investigating this would require the use of principled estimates or measurements of the fixation point within each analyzed image. In addition, once fixations and eye movements are being considered, it would be natural to extend the measurements into the temporal domain. This could provide new insights into how the temporal asymmetries between the ON and OFF pathways may contribute to differences in the motion input to cortex [16, 57].

Future work can also address the question of what the underlying geometric properties of natural scenes are that produce biases in visual cortical input. Addressing this question will require generating a 3D rather than a 2D synthetic scene model. For example, in future work we can test the hypothesis that shadows between objects produce more low spatial frequencies in the OFF pathway. This can be done by synthesizing 3D scenes and rendering them with and without directional light and shadows. However, the synthetic scenes must first be matched to natural scenes in terms of their material properties and distribution of 3D surfaces. Another potential direction for examining the sources of dark/bright biases is to determine if the magnitude of each bias correlates with any basic global image property, such as mean luminance. This approach would be advantageous because it can be performed on existing natural image datasets, however one would still be left to speculate as to which fundamental 3D scene properties produce the global image differences.

Recently, analogues of the visual ON and OFF domains—encoding positive and negative input states—have been identified in the olfactory and auditory systems [58, 59]. Future work can examine if similar adaptive asymmetries exist for these other sensory modalities as well.

### Conclusions

Previous statistical descriptions of the building-blocks of our visual world—small contours, regions of shading and contrast, three-dimensionality—have largely considered bright and dark features to be equivalent. Here, we have described the asymmetries between the statistics of brights and darks. We found that low spatial frequency image content is dominated by dark features. In addition, areas of high visual contrast are biased towards being dark, as are relatively distant features. We have also shown that a simple naturalistic image model can reproduce these biases in detail. This suggests that dark/bright asymmetries represent fundamental regularities of natural images and therefore do not arise from particularities of any specific image sets.

In addition, a basic visual computation—local light adaptation—contributes to the asymmetries by boosting contrast in dark image regions. Adaptation and normalization processes exist throughout the visual system, protecting against neuronal response saturation and allowing perceived contrast to be roughly invariant to light intensity [60]. In our synthetic image analysis, we showed that contrast normalization operations may interact with the 1/*f* ^*α*^ spatial frequency spectrum of natural images to boost low spatial frequency patterns in the OFF pathway. Thus, the dark/bright asymmetries are likely a pervasive property of visual input to the brain.

One key outcome of our analysis is to show that it does not make sense to directly connect natural scene image patterns in pixels to efficient and optimal encoding principles in visual cortex. We have demonstrated that the early stages of visual processing—which themselves are likely guided by efficiency [61]—alter the statistical patterns of visual features, and it is these patterns that must be driving the cortical encoding process.

Having performed these analyses, we can now propose a more comprehensive explanation for a body of recent work showing that primary cortical cells often violate the assumptions of the classic energy models used to describe them. We propose that many of the asymmetries in activity devoted to darks and brights in primary visual cortex—and even the visual system of flies [42]—reflect a specialization for processing the patterns of dark and bright input from the early visual pathways. While previous work has argued that dark dominance is overall adaptive for environmental input [25], we have shown here that highly specific patterns of visual features are reflected in this cortical specialization.

## Supporting Information

S1 File MATLAB code for simulating retinal ganglion cell responses to an image. (ZIP)

S1 Fig Probabilities and dark/bright ratios from a second set of natural scenes. (A-C) Probability densities are plotted as in Fig 3 for a set of images from a second dataset [27]. (C-D) Dark/bright ratios also plotted as in Fig 3. A few differences appear but the overall results are similar. Depth results are omitted because they were only available from a single dataset [19]
(EPS)

S2 Fig Dark/bright ratios across contrast-operator sizes. (A-D) Results are plotted as in Fig 3E–3H with the standard deviation of the central Gaussian (*σ*_*c*_) set to three different sizes in arcmin (see legend). Results for *σ*_*c*_ = 4 are the same as Fig 3. Across sizes ranging by a factor of 4, the qualitative dark/bright patterns are similar. As the size of the contrast operator increases, the spatial frequency asymmetry shifts towards lower spatial scales, because the contrast normalization area increases. The depth-dependent asymmetry also changes with size: the largest bias appears at the largest size.
(EPS)

S3 Fig Dark/bright ratios across contrast-operator shapes. (A-D) Results are plotted as in Fig 3E–3H with the ratio of the standard deviation of the surround Gaussian (*σ*_*s*_) to the central Gaussian (*σ*_*c*_) set to three different values (see legend). The standard deviation of the central Gaussian was fixed at 4 arcmin. Results for *σ*_*s*_/*σ*_*c*_ = 2 are the same as Fig 3. As in S2 Fig, when the size of the contrast operator increases, the spatial frequency asymmetry shifts towards lower spatial scales. This shift is rather substantial for the largest filter size (red line), for which *σ*_*s*_ is over 0.25 visual degrees.
(EPS)

S4 Fig Dark/bright ratios with an alternate definition of image contrast. (A-D) Results are plotted as in Fig 3E–3H, except that here local contrast is defined as the Weber contrast of each image pixel relative to a low-pass Gaussian filtered version of the image. The standard deviation of the low-pass Gaussian (*σ*) was set to three different sizes in arcminutes (see legend). Using this alternate definition of contrast, the overall dark/bright patterns are qualitatively similar to the main analysis. As in S2 Fig, it is clear that when the local normalization pool becomes relatively the large, the spatial frequency asymmetry is shifted.
(EPS)

S5 Fig Dark/bright ratios for multiple types of noise images. In addition to the white noise versus naturalistic noise comparison reported in the main analysis, we also compared the results for multiple classes of noise, each one containing an additional global feature of natural images (see Methods). This analysis uncovered which properties of natural images may lead to the dark dominance patterns. (A-D) Each line shows the results for a different type of noise image. Gaussian white (purple line) is the least naturalistic noise, and the dark dominance patterns are dissimilar to the natural scenes. Introducing a 1/*f* ^*α*^ spatial frequency distribution (orange line) begins to produce a spatial frequency bias similar to that of natural scenes. The patterns become more similar to natural scenes when positively skewed luminance values are added (red line). Adding in a cardinal orientation bias (blue line) recreates the very minor fluctuations in OFF dominance over orientation, and imposing a negative correlation between intensity and depth (yellow line) produces a similar far/dark dominance. The final class of noise is referred to in the main analysis as *naturalistic noise* because it has all of the global features necessary to reproduce the dark dominance patterns in natural scenes. The fact that these patterns can be closely matched by random phase noise images suggests that they arise from the global statistics of natural images, rather than specific geometric properties.
(EPS)
