# Supplementary figures and images for: Predicting Cortical Dark/Bright Asymmetries from Natural Image Statistics and Early Visual Transforms
Source: PLoS Comput Biol. 2015 May 28;11(5):e1004268. doi: 10.1371/journal.pcbi.1004268 (PMC4447361; doi:10.1371/journal.pcbi.1004268)

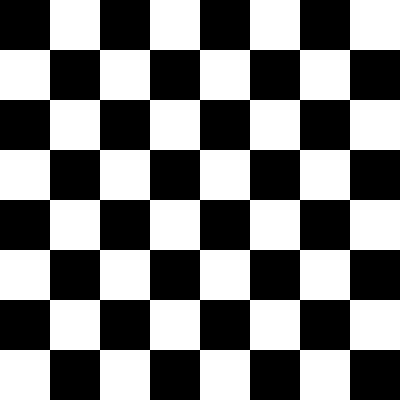

Supplement: S1 File — (ZIP) [file pcbi.1004268.s001.zip › OnOffCode/example_images/checker.png]

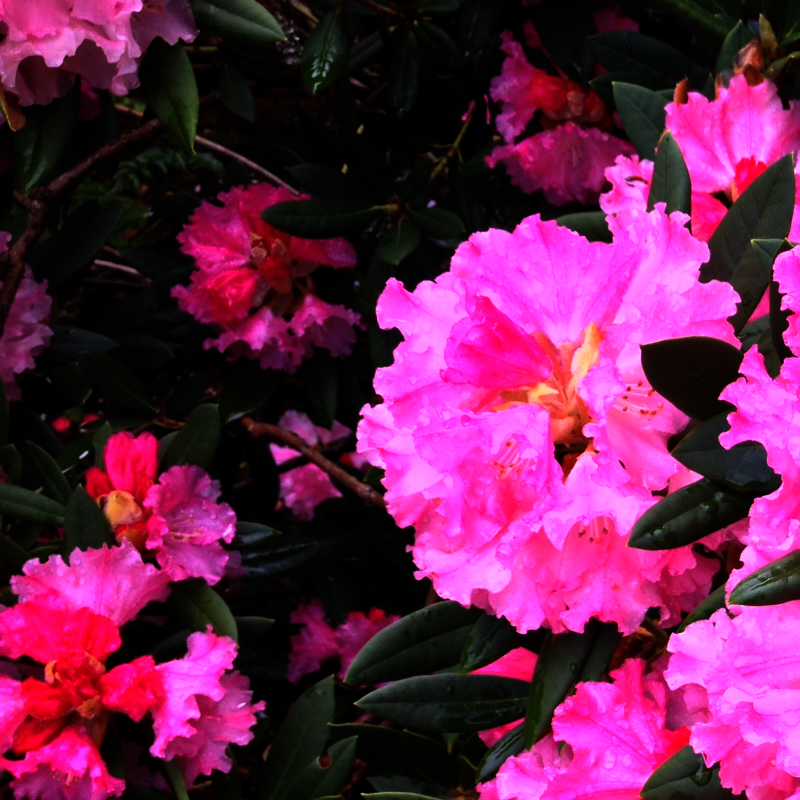

Supplement: S1 File — (ZIP) [file pcbi.1004268.s001.zip › OnOffCode/example_images/flowers.png]

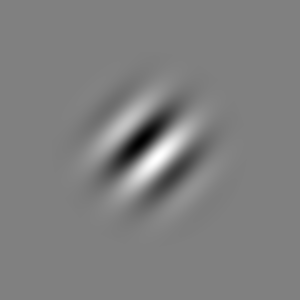

Supplement: S1 File — (ZIP) [file pcbi.1004268.s001.zip › OnOffCode/example_images/gabor.png]

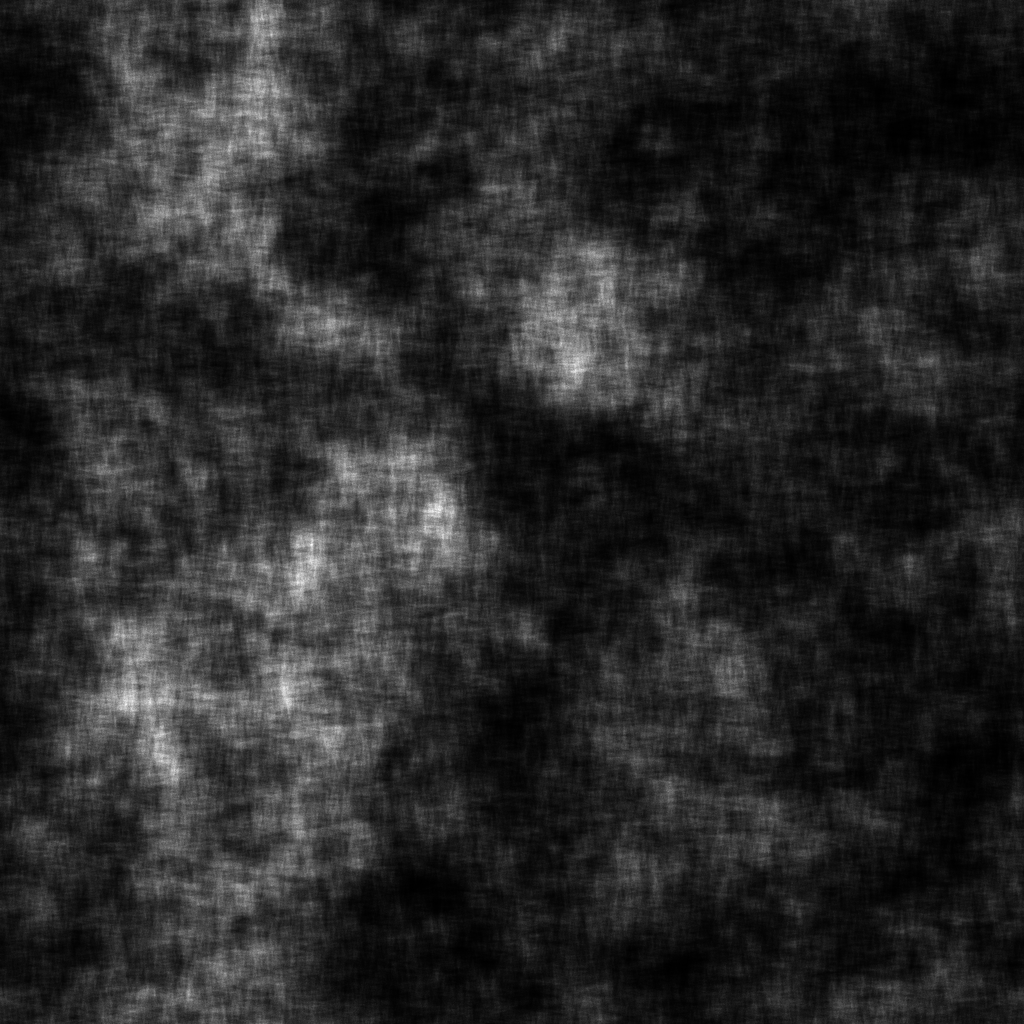

Supplement: S1 File — (ZIP) [file pcbi.1004268.s001.zip › OnOffCode/example_images/natural_noise.png]

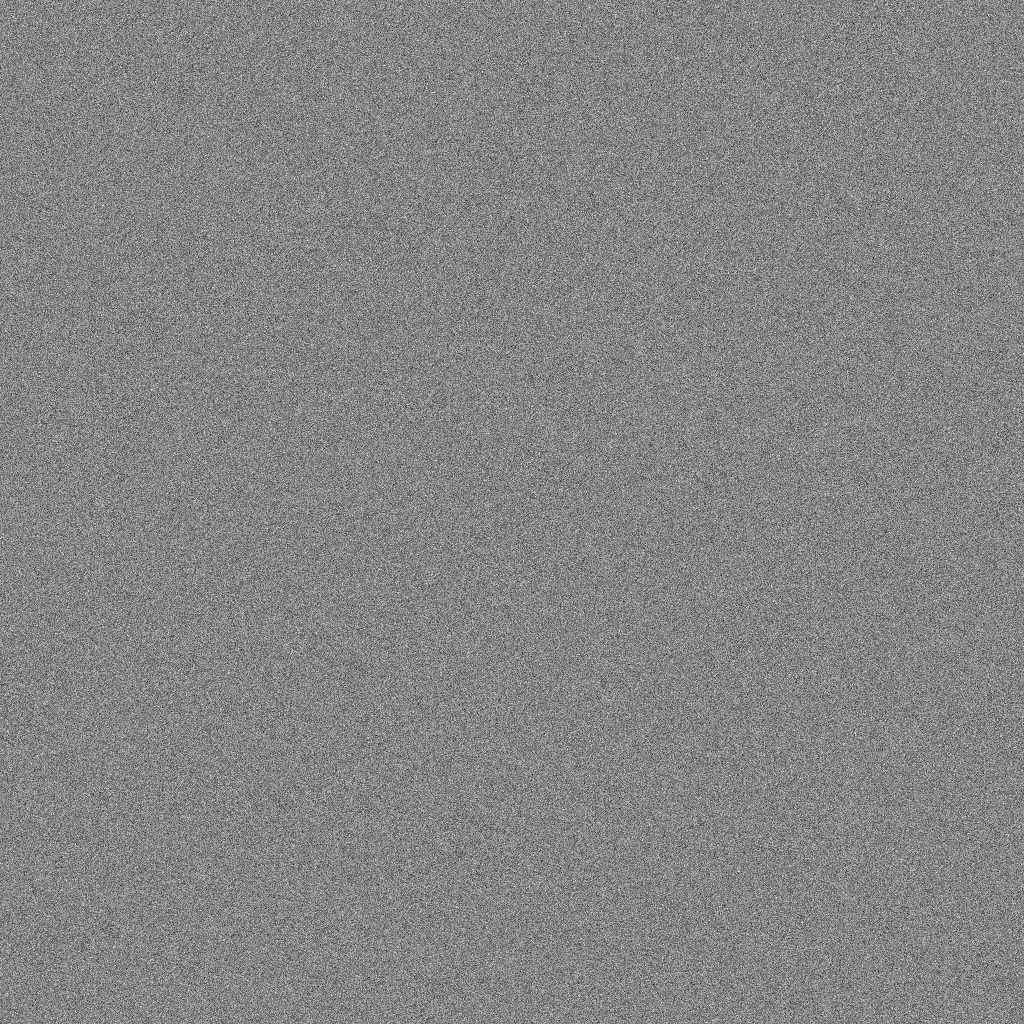

Supplement: S1 File — (ZIP) [file pcbi.1004268.s001.zip › OnOffCode/example_images/white_noise.png]
